# Identification of blood-activating components from Xueshuan Xinmaining Tablet based on the spectrum–effect relationship and network pharmacology analysis

**DOI:** 10.1039/c9ra09623j

**Published:** 2020-03-10

**Authors:** Jing Tan, Junli Liu, Han Wang, Ying Zhang, Hongqiang Lin, Zhongyao Wang, Hanrui Si, Yutong Zhang, Jinping Liu, Pingya Li, Kai Sun

**Affiliations:** School of Pharmaceutical Sciences, Jilin University Fujin Road 1266 Changchun 130021 Jilin China liujp@jlu.edu.cn lipy@jlu.edu.cn thomassk@jlu.edu.cn +86-431-85619803; Research Center of Natural Drug, Jilin University Changchun 130021 China; The First Hospital of Jilin University Changchun 130021 Jilin China

## Abstract

With the aim of identifying the active components of Xueshuan Xinmaining Tablet (XXT) and discussing the potential mechanism involved, the relationship between HPLC fingerprints and its blood-activating effect were established by multivariate statistical analysis, including gray relational analysis (GRA) and partial least squares regression analysis (PLSR). Network pharmacology was used to predict the potential mechanism based on the identified active components. GRA and PLSR analysis showed close correlation between the HPLC fingerprints and blood-activating activity, and peaks P1, P3, P11, P15, P22, P34, P36, P38 and P39 might be potential anti-blood stasis components of XXT. The pharmacological verification showed that salvianic acid A (P1), rutin (P3), ginsenoside Rg_1_ (P11) and Rb_1_ (P22), cinobufagin (P36), and tanshinone I (P38) and IIA (P39) had significant blood-activating effects. Based on these seven active compounds, network pharmacology analysis indicated that the anti-blood stasis effect of XXT might be closely related to TNF, PI3K-Akt and NF-κB signaling pathways. The spectrum–effect relationship of XXT was successfully established in this study. The blood-activating components and the anti-blood stasis mechanism were revealed and predicted. These findings could also be beneficial for an exploration of the active components of TCM.

## Abstract 2

To identify active components of XXT and discuss the potential mechanism, the relationship between HPLC fingerprints and blood-activating effects were established by GRA and PLSR, and the mechanism was discussed by the network pharmacology analysis.

## Graphical abstract

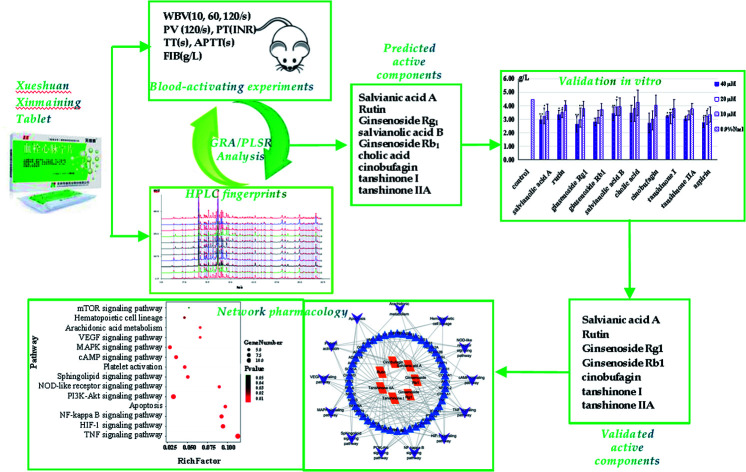

## 1 Introduction

Xueshuan Xinmaining Tablet (XXT), a Chinese traditional compound medicine composed of ten herbs, possesses the activities of promoting blood circulation and removing blood stasis.^1^ It is recorded in the *Chinese Pharmacopoeia* (2015 edition) and has been clinically applied for the treatment of cerebral thrombosis and coronary heart disease for at least fifteen years.^2,3^ In our previous studies, we have reported that XXT could treat blood stasis through regulating related genes and proteins or endogenous metabolite pathways.^1,4,5^ The multi-target mechanism is closely related to the variation in the chemical components contained. We have reported the comprehensive component screening of XXT based on UPLC/Q-TOF-MS. The results showed that XXT was rich in chemical constituents of different structural types.^6^ Additionally, the contents of salvianolic acid B, rutin, ferulic acid, and ginsenoside Re and Rg_1_ were determined in the national standard of XXT. However, whether these ingredients are the blood-activating substances is not quite certain.

Fingerprints are widely recognized as a quality control method worldwide.^7,8^ The World Health Organization (WHO, 2007) has approved the use of fingerprints to evaluate the quality of natural drugs or traditional Chinese medicine (TCM). Fingerprints, with the advantages of convenience and a large amount of information, could systematically mark the chemical composition of TCM.^9^ The UPLC-PDA fingerprint of XXT, marked with 28 common peaks in 280 nm, has been established to assess the quality consistency of XXT.^10^ However, the fingerprints only focused on the chemical characteristics, but gave little information about the components' therapeutic effects. The components in XXT related to the anti-blood stasis effect have not been identified.

The spectrum–effect relationship was established by linking the peaks in the fingerprints of TCM with specific bioactivity to screen the active components in TCM.^11^ Multivariate statistical methods, including partial least squares regression analysis (PLSR) and gray relational analysis (GRA), have been commonly applied to analyze the regression coefficients, variable importance in projection (VIP) contribution, and their correlation.^12–14^ As a result, the active compounds closely related to a specific effect could be effectively screened for further pharmacological activity evaluation *in vitro* or *in vivo*.

With the development of bioinformatics, network pharmacology, as a more comprehensive approach for integrating compound–target–pathway interactions from a molecular to a systematic level, is evolving as a Frontier research field in drug discovery and development. The boom in network pharmacology has prompted more researchers to elucidate the possible mechanisms of natural products in recent years.^15,16^

In this study, the blood-activating components in XXT were predicted by analysis of the spectrum–effect relationship for the chemical characteristic peaks and the anti-blood stasis effect. In view of the uniqueness of the manufacturer, and the good similarity and stability of chemical components in the XXT fingerprint,^10^ ten different polar solvent extracts of XXT were used to establish HPLC fingerprints. Then, a pharmacological test *in vitro* was performed to verify the predicted results. Finally, the possible targets and signaling pathways of the blood-activating components were predicted by network pharmacology. This study identified the anti-blood stasis components in XXT, which could be beneficial for the quality control of XXT and the exploration of blood-activating components.

## 2 Experiments

### 2.1 Instruments, materials, and animals

#### 2.1.1 Instruments

Agilent ZORBAX SB C_18_ column (4.6 mm × 250 mm, 5 μm; Agilent Technologies, MA, USA); Waters 1525 HPLC, Waters 2998 Diode Array Detector (American Waters); FA1104N One–tenth Electronic Analysis Balance (Shanghai Jinghua Technology Instrument Co., Ltd.); R201D Constant Temperature Water Bath and Rotary Evaporator (Shanghai Yukang Science and Education Equipment Ltd.); KQ3200V Ultrasonic Cleaner (150 W, 40 kHz, Kunshan Ultrasonic Instrument Co., Ltd.). SC40 (LG-PABER-I) semi-automatic coagulation factor analyzer Taizhou Steellex (Biotechnology Co. Ltd. China); KES-900B blood rheometer (Wuxi Kangersheng Electronic Instrument Co. Ltd. in China); LBY-N6 cone–plate blood viscometer (Precil Co., Ltd. Beijing, China) and XN2000 hematology analyzer (Sysmex Corporation, Kobe, Japan).

#### 2.1.2 Materials

XXT (Jilin Huakang Pharmaceutical Co., Ltd. Jilin, China); heparin sodium (YM Biological Technology Co., Ltd. Shanghai, China); epinephrine (Tianjin Pharmaceuticals Group Co., Ltd. Tianjin, China); chloral hydrate (Biosharp Co., Ltd. Shenyang, China); acetonitrile suitable for HPLC (Fisher Chemical Company, Shanghai, China); deionized water purified using a Milli-Q water purification system (Millipore, Billerica, MA, USA); Sysmex coagulant analyzer (Sysmex Corporation, Japan); phosphoric acid (Beijing Chemical Works, Beijing, China). Standards of tanshinone IIA, ginsenoside Rg_2_, -Rd, -Rf, -Rh_2_, -Rb_2_, -Rb_3_, -Rg_1_, -Re, -Rb_1_, rutin, quercetin, cinobufagin and resibufogenin (National Institutes for Food and Drug Control, Beijing, China); hyodeoxycholic acid, cholic acid, salvianic acid A, salvianolic acid B and tanshinone I (Sichuan Wei Keqi Biotechnology Co., Ltd., Sichuan, China); ginsenoside Rh_1_, -Rc, -Rg_3_ and -F_2_ were prepared by the laboratory.

#### 2.1.3 Animals

Wistar rats (male, 200 ± 20 g) were purchased from the Animal Laboratory Center of the Basic Medical College of Jilin University (Changchun, China) (Certificate No. SCXK-(Ji) 2018-0003) and used for experiments after a week of adaptive feeding. The rats were divided randomly into 13 groups and assigned to PC polycarbonate mouse cages (53.5 cm × 39.0 cm × 20.0 cm) (*n* = 10 for each group per cage), then allowed standard diets and water *ad libitum*. The rats were kept in a controlled environment (temperature: 20 ± 2 °C; relative humidity: 50 ± 10%) with a 12 h dark/light cycle. All animal procedures were performed in accordance with the Guidelines for Care and Use of Laboratory Animals of Jilin University and approved by the Animal Ethics Committee of Jilin University. The approval number was 201904108.

### 2.2 HPLC fingerprints

#### 2.2.1 HPLC conditions

Column temperature, 40 °C; detection wavelength, 203 nm; injection volume, 10 μL; flow rate, 1.0 mL min^−1^; mobile phase acetonitrile (A)–0.1% phosphoric acid aqueous solution (B) with the following gradient elution: 0–10 min, 5% A; 10–20 min, 5% → 10% A; 20–35 min, 10% → 15% A; 35–55 min, 15% → 20% A; 55–75 min, 20% → 28% A; 75–115 min, 28% → 35% A; 115–140 min, 35% → 40% A; 140–190 min, 40% → 60% A.

#### 2.2.2 Preparation of solutions

##### 2.2.2.1 Reference standard solutions

Certain amounts of salvianolic acid B, salvianic acid A, rutin, quercetin, ginsenoside Rg_1_, -Re, -Rf, -Rb_1_, -Rg_3_, -Rh_1_, -Rc, -Rg_2_, -F_1_, -Rb_2_, -Rb_3_, -Rd, -F_2_, cholic acid, cinobufagin, resibufogenin, tanshinone I and tanshinone IIA were dissolved in methanol to obtain the mixed reference standards solutions (the concentration of each reference standard was 0.1 mg mL^−1^).

##### 2.2.2.2 Sample solutions

The pulverized XXT sample was sieved (Chinese National Standard Sieve No. 3, R40/3 series) to obtain a homogeneous powder. Then each fine powder was accurately weighed (50 g). Each powder was extracted respectively with different polarity solvents {S_1_: *n*-butanol; S_2_: methanol; S_3_: methanol–dichloro (1 : 1); S_4_: 70% methanol; S_5_: ethanol–acetone (1 : 1); S_6_: ethyl acetate; S_7_: ethanol–ethyl acetate (1 : 1); S_8_: ethanol; S_9_: methanol–acetone (1 : 1); S_10_: 50% acetone} in an ultrasonic bath at 40 °C three times (1 h each time). After being filtered, the extraction solutions were combined, concentrated, and evaporated to dryness. The desiccated extracts were then respectively dissolved in methanol to obtain the solutions (each was 20 mg mL^−1^), mixed well, and filtered (0.45 μm).

#### 2.2.3 Validation of method

The optimized method was validated by evaluating the precision, repeatability and stability. Precision was evaluated by successively analyzing the six test solutions from the S_4_ sample. Six replicates of the S_4_ sample were successively assessed to evaluate the repeatability. The stability was determined by analysis at different time intervals (0, 2, 4, 8, 16, and 24 h) in a day. Furthermore, the precision, repeatability and stability were expressed as the relative standard deviation (RSD) of each peak area (PA) of each sample.

#### 2.2.4 Evaluation of HPLC fingerprints

##### 2.2.4.1 SA

Each sample solution (S_1_–S_10_) and the reference standard solutions were injected into the HPLC. Chromatograms showing the retention time (*t*_R_) and the peak area (PA) were obtained. According to the Similarity Evaluation System for Chromatographic Fingerprint of TCM (version 2012A; Beijing, China), the fingerprints were automatically matched and established.^17^ Chromatograms of S_1_–S_10_ samples, including PA and *t*_R_, were put into the Analytical Instrument Association (AIA) form (*.cdf). Subsequently, a reference fingerprint was automatically generated by comparing S_1_–S_10_ samples based on the median method, and the similarity (SA) between the chromatogram of the reference fingerprint and each sample chromatogram was calculated with the software.

##### 2.2.4.2 HCA

Hierarchical cluster analysis (HCA), a multivariate statistical analysis technique to measure dissimilarity or similarity, is commonly used to sort samples into clusters.^18^ The common PAs of the samples were used as features, and the HCA of the S_1_–S_10_ samples was established from the squared Euclidean distance (metric) and the between-groups linkage method (the amalgamation rule) with SPSS statistics software (SPSS 19.0, SPSS Inc., Chicago, IL, USA).^19^

### 2.3 Blood-activating experiments

#### 2.3.1 Sample preparation

XXT extract samples were prepared according to the method described in Section 2.2.2. Significantly, the desiccated extracts were suspended in distilled water instead of being dissolved in methanol. The concentrations of XXT extracts were calculated from the amount of XXT, and the final concentrations were equivalent to 70.0 mg mL^−1^ of XXT. A Buchang Naoxintong Capsule (BCN) was also suspended in distilled water to produce a solution containing 70.0 mg per mL BCN. So 10 kinds of XXT extract and BCN test solutions were prepared according to a dosage of 0.7 g kg^−1^.^1^

#### 2.3.2 Grouping, modeling, and administration

After one week of adaptive feeding, 130 male Wistar rats were randomly divided into 13 groups (10 rats per group): (i) normal (N) group, (ii) model (M) group, (iii) BCN positive control group, and (iv–xiii) the XXT extract (S_1_–S_10_) groups. Both i and ii groups were administered with distilled water. All groups were gastrointestinally administered at 10 mL kg^−1^ once a day for 8 days. On day 1 to day 6, half an hour after each administration, the ii–xiii groups were all placed in ice water (0–1 °C) for 5 min. On day 7, 30 minutes after intragastric administration, the ii–xiii groups were injected subcutaneously with 0.1% adrenaline (1 mg kg^−1^) twice to establish an acute blood stasis model.^20^ The first dose was 1 mg kg^−1^, and the second dose was reduced to 0.8 mg kg^−1^ at an interval of 4 hours. Two hours after the first dose of adrenaline, the rats in the ii–xiii groups were also immersed in an ice bath for 5 min.

#### 2.3.3 Determination of hemorheology and coagulation indicators

In order to assess the model in rats and the effect of XXT extracts, the hemorheology indexes (whole blood viscosity: WBV; plasma viscosity: PV) and coagulation indicators (thrombin time: TT; prothrombin time: PT; activated partial thromboplastin time: APTT; fibrinogen: FIB) were evaluated.^21–23^

On the night of day 7, all the rats were fasted for 12 h, but water was allowed. On day 8, 30 min after administration, the rats were anesthetized by an intraperitoneal injection of 10% chloral hydrate (3 mL kg^−1^). Blood was drawn from the abdominal aorta and was kept in tubes containing heparin sodium (20 U mL^−1^). Some of the whole blood was used to measure the whole blood viscosity (WBV). The WBV was determined (at shear rates of 10, 60, and 120/s) at 37 °C. Plasma, used to determine PV, TT, PT, APTT, and FIB, was obtained from the remaining whole blood by centrifugation at 3000 rpm for 10 min. PV was measured at a shear rate of 120/s. TT, PT, APTT, and FIB were determined with an SC 40 (LG-PABER-I) coagulation analyzer with commercial kits, in accordance with the manufacturers' instructions. The calibration curves of PT (*x*) and solidification time (*y*), FIB (*x*) and solidification time (*y*) were established. TT was tested by incubating 100 μL of plasma at 37 °C for 3 min, followed by the addition of 100 μL of thrombin agent. PT was examined by incubating 50 μL of plasma for 3 min at 37 °C, followed by the addition of 100 μL of thromboplastin agent. APTT was evaluated by incubating 50 μL of plasma with 50 μL pf APTT-activating agents for 3 min at 37 °C, followed by the addition of 50 μL of CaCl_2_. FIB was assessed by incubating 10 μL of plasma with 90 μL of imidazole buffer at 37 °C for 3 min, followed by the addition of 50 μL of FIB agent. All hemorheological indexes and coagulation function indexes were determined within 3 h after blood collection.

#### 2.3.4 Data analysis

All quantitative data were presented as mean ± standard derivation (SD) calculated with GraphPad Prism 6.0 software (GraphPad Software, CA). Multiple comparisons were analyzed by one-way analyses of variance (ANOVA). A Student's *t*-test was carried out by comparing two groups. A *p*-value < 0.05 was considered statistically significant.

### 2.4 Spectrum–effect relationship analysis

#### 2.4.1 GRA

Gray relational analysis (GRA), expressing the strengths of relationships between factors, has been widely applied for TCM studies of spectrum–effect relationships.^24,25^ Compared with other statistical methods, the gray correlation analysis, with lower requirements on sample data, possesses the advantages of requiring a small sample size and amount of computation, and has been applied widely. The principle of GRA is to utilize the degree of similarity between two series of curve geometries to determine a correlation based on assessing the influence of factors on the index.^26,27^ Its basic method is to use the pharmacodynamic index of traditional Chinese medicine as the reference sequence and the characteristic peak area shared by the fingerprints as the comparison sequence. According to the gray correlation between each factor of the comparison sequence and the reference sequence, the “contribution” of each common characteristic peak to the efficacy is determined.^28,29^ The higher the gray relational grade is, the greater the correlation. In this study, GRA was performed using DPS 7.05 software (Data Processing system, China).

#### 2.4.2 PLSR

Partial least squares regression analysis (PLSR), a multivariate statistical regression model,^30^ could effectively solve the multicollinearity between a set of dependent variables (*y*) from a large set of independent variables (*x*).^31^ In this study, the *x* matrix was composed of PA, and the *y* vector was constructed with APTT and the levels of WBV (10/s). The PLSR model was established with SIMCA-P software (SIMCA-P13.0, Umetrics, Umeå, Sweden).^32^ The relative influence of the independent variables on the dependent variables was reflected by the regression coefficient.

### 2.5 Pharmacological test

The predicted components related to the blood-activating activity of XXT were identified with reference standards. The anticoagulant activity of the predicted components of different concentrations (10, 20, and 40 μM) on normal rat plasma was determined by the method described in Section 2.3.3.^33,34^ The bioactive components were finally screened out with this *in vitro* test.

### 2.6 Network pharmacology

Network pharmacology, an application of systems biology, is applied to predict the complex mechanism of action of TCM formulas.^14,35^ It could be used to construct an entire drug–target interaction network and to predict the core targets and pathways involved. It is more efficient to clarify how multiple components interact with core targets by intersecting with the disease target database. In this study, the aforementioned screened active components were used as “candidate compounds” for network pharmacological analysis. The interaction network between active components and a blood stasis related target was then presented, and the potential involved pathways were also predicted.

#### 2.6.1 Construction of networks

##### 2.6.1.1 The compound–target network

To collect the targets of the active components, some databases, such as TCMSP (http://lsp.nwsuaf.edu.cn/tcmsp.php), SymMap (http://www.symmap.org), TCM-MeSH (http://mesh.tcm.microbioinformatics.org/), ETCM (http://www.nrc.ac.cn:9090/ETCM/), and BATMAN–TCM (http://bionet.ncpsb.org/batman-tcm), were used. The active component–target interaction map was then generated with Cytoscape 3.6.0 software (http://www.cytoscape.org/).

##### 2.6.1.2 The target–target network

Blood stasis-associated target proteins were collected by using “blood stasis”, “coagulation of blood”, “blood clotting”, “blood viscosity”, “blood rheology”, “blood thickening”, “thrombus”, “thrombosis”, “thrombophilia”, “thromboembolism”, “venous thrombosis”, “cerebral thrombosis”, and “thromboses” as the keywords for a search in the OMIM database (http://omim.org/), DisGeNET database (http://www.disgenet.org/), and the PharmGKB database (http://geneticassociationdb.nih.gov/). By intersecting with the targets of screened compounds, the core targets were then obtained. The protein–protein interaction (PPI) network was constructed with the STRING database (String, https://string-db.org/).

#### 2.6.2 GO function enrichment and KEGG pathway enrichment analysis

##### 2.6.2.1 GO

We enriched the overexpressed GO (gene ontology) terms of the obtained target networks. The gene symbol IDs of the core targets were transformed to gene ensemble IDs by using the Ensembl database (https://asia.ensembl.org/). Gene ensemble ID files were then uploaded to the OmicShare database (https://www.omicshare.com/) for GO enrichment analysis.

##### 2.6.2.2 KEGG

The target-enriched disease pathways were obtained by Kyoto Encyclopedia of Genes and Genomes (KEGG) pathway enrichment analysis of core targets using the DAVID database (https://david.ncifcrf.gov/). The OmicShare database (http://omicshare.com/) was used to visualize the result.

## 3 Results

### 3.1 Results of HPLC fingerprints

#### 3.1.1 Method validation

The *t*_R_ and PA of common peaks were both calculated to assess the precision, repeatability, and stability. The results demonstrated that the relative standard deviation (RSD) of the precision (*t*_R_: 0.26–1.54%; PA: 0.32–1.85%), repeatability (*t*_R_: 0.25–1.36%; PA: 0.12–1.76%), and sample stability (*t*_R_: 0.07–1.27%; PA: 0.18–1.42%) were all lower than 3%. All results illustrated that the established HPLC analysis method was valid and suitable for sample analysis.

#### 3.1.2 Results of HPLC fingerprints

**Fig. 1:**
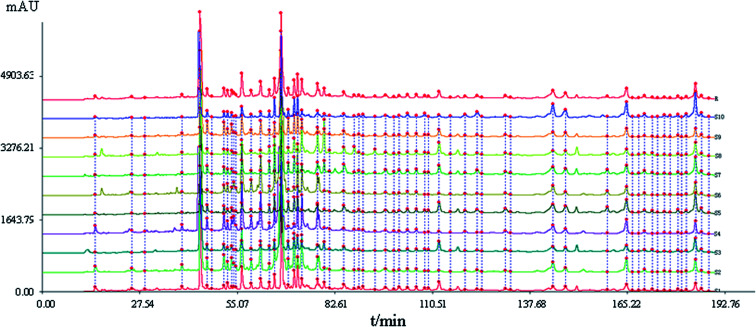
HPLC fingerprints of samples S_1_–S_10_ of Xueshuan Xinmaining Tablet (XXT). (S_1_–S_10_: 10 kinds of extract with different polar solvents; R: the reference fingerprint).

**Fig. 2:**
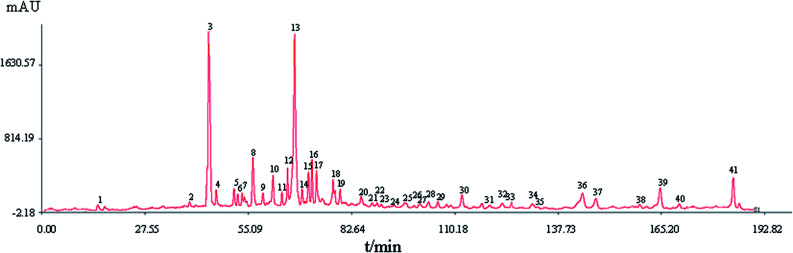
The reference fingerprint of XXT.

HPLC fingerprints of 10 batches of S_1_–S_10_ samples are shown in Fig. 1. The automatically generated reference fingerprint (R) is shown in Fig. 2.

**Fig. 3:**
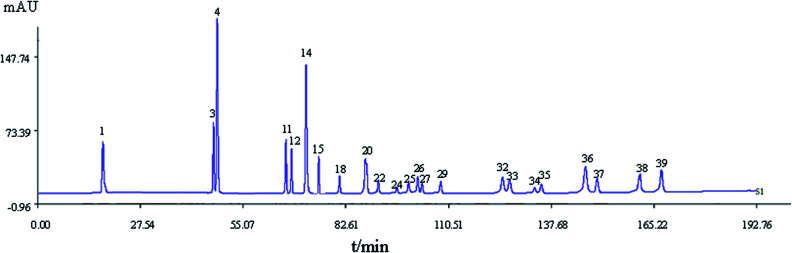
The HPLC spectrum of a mixed standard reference. 1: salvianic aid A; 3: rutin; 4: quercetin; 11, 12, 14, 18, 20, 22, 24, 25, 26, 27, 29, 32, 33: ginsenoside Rg_1_, -Re, -Rf, -Rh_1_, -Rg_2_, -Rb_1_, -Rc, -Rb_2_, -Rb_3_, -Rd, -Rg_3_, -F_2_, -Rh_2_; 15: salvianolic acid B; 34: cholic acid; 35: hyodeoxycholic acid; 36: cinobufagin; 37: resibufogenin; 38: tanshinone I; 39: tanshinone IIA.

A total of 41 peaks with good segregation and PA greater than 1 000 000 were selected as the “common peaks” from consecutive peaks. Rutin (Peak 3), identified by comparison with reference standards with *t*_R_ at 44.4 min, was selected as the reference peak to calculate the relative *t*_R_ and relative PA of the other 40 common peaks. In accordance with the mixed reference standard HPLC spectrum shown in Fig. 3, 23 characteristic peaks, P1, P3, P4, P11, P12, P14, P18, P20, P22, P24, P25, P26, P27, P29, P32, P33, P15, P34, P35, P36, P37, P38, and P39, were identified as salvianic acid A, rutin, quercetin, ginsenoside Rg_1_, -Re, -Rf, -Rh_1_, -Rg_2_, -Rb_1_, -Rc, -Rb_2_, -Rb_3_, -Rd, -Rg_3_, -F_2_, -Rh_2_, salvianolic acid B, cholic acid, hyodeoxycholic acid, cinobufagin, resibufogenin, tanshinone I, and tanshinone IIA, respectively. The variance coefficients for almost all common peaks were greater than 35%. This is due to the diversity in the contents of constituents contained in different samples extracted with different polar solvents.

##### 3.1.2.1 SA

The similarity values between the entire chromatogram of the S_1_–S_10_ samples and the reference fingerprint were assessed, and their correlation coefficients were 0.948, 0.904, 0.986, 0.917, 0.974, 0.815, 0.986, 0.955, 0.987, and 0.963, respectively.

##### 3.1.2.2 HCA

**Fig. 4:**
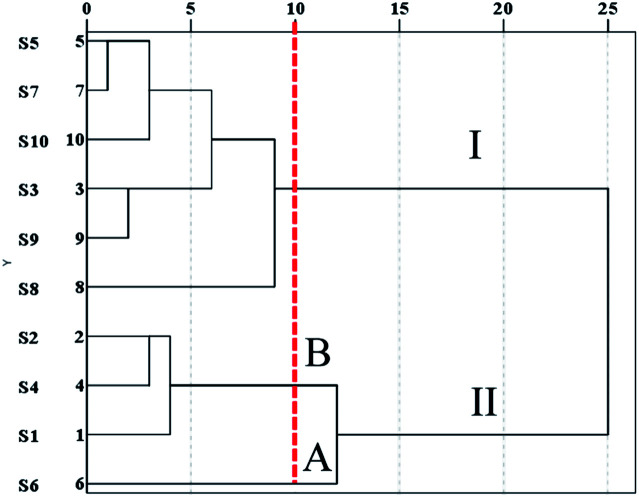
Hierarchical clustering analysis of XXT samples.

To further evaluate the results of the similarity analysis, HCA was performed. As shown in Fig. 4, the S_1_–S_10_ samples were obviously separated into two main clusters: S_5_, S_7_, S_13_, S_9_, and S_8_ in cluster I, and the other samples in cluster II. Cluster II was further separated into two subgroups: subgroup A (S_6_) and subgroup B (S_2_, S_4_, S_1_). The results demonstrated that the chemical components varied from S_1_ to S_10_, which was confirmed by the similarity analysis.

### 3.2 Results of blood-activating activity

The effects of S_1_–S_10_ on WBV, PV, TT, APTT, PT, and FIB are shown in Table 1.

**Table 1:** The whole blood viscosity (WBV), plasma viscosity (PV), prothrombin time (PT), thrombin time (TT), activated partial thromboplastin time (APTT), and fibrinogen (FIB) results from rats (*n* = 10)^a^

Group	WBV (mPa s; expressed at high, medium and low shear rates)			PV (mPa s; 120/s)	PT (INR)	TT (s)	APTT (s)	FIB (g L^−1^)
	10/s	60/s	120/s					
N	6.9 ± 0.9	5.6 ± 0.4	4.8 ± 0.4	1.27 ± 0.08	27 ± 2	41 ± 3	22 ± 2	1.7 ± 0.8
M	8.0 ± 0.5^##^	6.1 ± 0.4^#^	5.6 ± 0.4^#^	1.8 ± 0.3^##^	24.1 ± 0.7^##^	35 ± 1^##^	18.3 ± 1.0^##^	3.7 ± 0.5^##^
BCN	7.0 ± 0.4**	5.4 ± 0.5**	5.0 ± 0.4*	1.4 ± 0.2**	26.2 ± 0.7**	40 ± 1**	20 ± 2**	2.4 ± 0.8*
S_1_	7.6 ± 0.4	5.6 ± 0.4*	5.2 ± 0.4*	1.51 ± 0.07*	23.8 ± 0.8	36.2 ± 0.2	18.6 ± 0.3	2.2 ± 0.8**
S_2_	7.3 ± 0.8*	5.6 ± 0.5*	5.2 ± 0.5*	1.4 ± 0.4*	24.8 ± 0.7*	38 ± 1**	20.1 ± 0.6**	2.1 ± 0.8**
S_3_	7.2 ± 0.5**	5.7 ± 0.4	4.9 ± 0.5**	1.4 ± 0.5*	25.1 ± 0.8*	38 ± 1**	20 ± 2**	2.9 ± 0.8*
S_4_	6.8 ± 0.4**	5.4 ± 0.4**	4.9 ± 0.4**	1.3 ± 0.3**	26.7 ± 0.7**	41 ± 1**	21 ± 1**	2.0 ± 0.8**
S_5_	6.9 ± 0.4**	5.5 ± 0.5*	5.0 ± 0.4**	1.4 ± 0.4*	26.3 ± 0.7**	40 ± 1**	20 ± 2*	2.0 ± 0.8**
S_6_	7.3 ± 0.6**	5.7 ± 0.3	5.2 ± 0.2*	1.5 ± 0.3*	24.9 ± 0.7*	38 ± 1**	18.8 ± 0.6*	2.1 ± 0.8**
S_7_	6.9 ± 0.5**	5.4 ± 0.5**	5.0 ± 0.5**	1.3 ± 0.3**	26.2 ± 0.6**	40 ± 1**	21.0 ± 0.6**	2.0 ± 0.8**
S_8_	7.1 ± 0.3**	5.7 ± 0.3*	5.2 ± 0.3*	1.4 ± 0.3**	25.3 ± 0.8**	39 ± 1**	21 ± 2**	2.1 ± 0.8**
S_9_	7.3 ± 0.6**	5.7 ± 0.6	5.2 ± 0.5	1.4 ± 0.1**	25.0 ± 0.7*	38 ± 1**	21 ± 1**	2.1 ± 0.8**
S_10_	7.3 ± 0.5**	5.7 ± 0.7	5.1 ± 0.5*	1.4 ± 0.3*	24.9 ± 0.7*	38 ± 1**	20 ± 2**	2.1 ± 0.8**

a Note: the data represent *x̄* ± s; compared with the M group, *p < 0.05; **p < 0.01. Compared with the N group, ^#^*p* < 0.05; ^##^*p* < 0.01.

The levels of WBV (10, 60, and 120/s) and PV of the M group were significantly increased compared with the N group (*p* < 0.05, *p* < 0.01). While the levels of WBV and PV of the S_1_–S_10_ groups were notably lower than those of the M group (*p* < 0.05, *p* < 0.01). The S_4_ and S_7_ groups exhibited the best effect on both indexes (*p* < 0.01). The BCN group could significantly reduce PV and the low, medium and high share rates of WBV (*p* < 0.05, *p* < 0.01).

In the model rats, TT and APTT were significantly shortened (*p* < 0.01), the content of PT was prominently decreased (*p* < 0.01), and the level of FIB was significantly increased (*p* < 0.01). TT and APTT were remarkably prolonged, the content of PT was significantly increased, and the contents of FIB were considerably reduced in the S_1_–S_10_ groups as well as in the BCN group (*p* < 0.05, *p* < 0.01).

It was demonstrated that different batches of XXT samples exhibited a noticeable blood-activating effect on the acute blood stasis model rats. However, considerable differences in the levels of WBV (10/s) and APTT between different batches of XXT samples were found by the ANOVA analysis, which revealed that the contents of active components in each kind of XXT extract were different to some extent.

### 3.3 Spectrum–effect relationship analysis

#### 3.3.1 GRA

To evaluate the spectrum–effect relationship, the correlation between the PA of 41 common peaks in the HPLC fingerprints and WBV (10/s) and APTT was calculated. The gray relational grades are listed in Table 2. The results revealed that the increase in PA contributed to a decrease in low shear rates (10/s) and APTT, following a descending order of peaks P34 > P27 > P1 > P38 > P22 > P11 > P15 > P36 > P41 > P6 > P39 > P3 and P38 > P34 > P1 > P28 > P15 > P36 > P22 > P27 > P41 > P3 > P11 > P29 > P39 (*r* > 0.80), respectively, which indicated that these peaks have potential strong blood-activating bioactivities.

**Table 2:** Correlation coefficient analysis between peaks and WBV and APTT

Peak no.	WBV (mPa s^−1^; 10/s)	APTT (s)	Peak no.	WBV (mPa s^−1^; 10/s)	APTT (s)	Peak no.	WBV (mPa s^−1^; 10/s)	APTT (s)
1	0.82	0.84	15	0.81	0.82	29	0.78	0.80
2	0.70	0.69	16	0.73	0.72	30	0.68	0.68
3	0.80	0.81	17	0.67	0.67	31	0.78	0.79
4	0.76	0.77	18	0.78	0.77	32	0.69	0.70
5	0.76	0.76	19	0.72	0.74	33	0.77	0.77
6	0.80	0.78	20	0.75	0.75	34	0.83	0.84
7	0.71	0.71	21	0.72	0.72	35	0.72	0.74
8	0.68	0.69	22	0.81	0.81	36	0.80	0.82
9	0.72	0.70	23	0.74	0.73	37	0.79	0.79
10	0.62	0.62	24	0.68	0.68	38	0.82	0.84
11	0.81	0.80	25	0.74	0.74	39	0.79	0.80
12	0.79	0.80	26	0.72	0.74	40	0.78	0.79
13	0.74	0.74	27	0.82	0.81	41	0.80	0.81
14	0.78	0.79	28	0.81	0.84			

#### 3.3.2 PLSR

Using SIMCA P+ 11 software, the PAs of 41 common peaks were selected as independent variables (*x*_1_–*x*_41_), and WBV (L, 10/s) and APTT (s) were selected as the dependent variables (*y*_1_ and *y*_2_, respectively). With PLSR, when *R*^2^ reached a maximum (0.849 for WBV and 0.671 for APTT), for WBV, the regression equation was: *y*_1_ = 0.000001 − 0.095280*x*_1_ + 0.000081*x*_2_ − 0.051383*x*_3_ − 0.030580*x*_4_ − 0.018198*x*_5_ − 0.000033*x*_6_ − 0.018723*x*_7_ − 0.006288*x*_8_ − 0.016949*x*_9_ − 0.011640*x*_10_ − 0.048024*x*_11_ − 0.039335*x*_12_ − 0.014598*x*_13_ − 0.018438*x*_14_ − 0.103297*x*_15_ − 0.009129*x*_16_ − 0.018413*x*_17_ − 0.052504*x*_18_ − 0.019004*x*_19_ − 0.034401*x*_20_ − 0.025763*x*_21_ − 0.062268*x*_22_ − 0.003126*x*_23_ − 0.034273*x*_24_ − 0.049758*x*_25_ − 0.079793*x*_26_ − 0.032436*x*_27_ − 0.065212*x*_28_ − 0.057417*x*_29_ − 0.007182*x*_30_ − 0.034690*x*_31_ − 0.024183*x*_32_ − 0.037980*x*_33_ − 0.065510*x*_34_ − 0.031718*x*_35_ − 0.064903*x*_36_ − 0.031920*x*_37_ − 0.104891*x*_38_ − 0.046858*x*_39_ − 0.064089*x*_40_ − 0.041304*x*_41_. For APTT, the regression equation was: *y*_1_ = 0.850803 + 0.007908*x*_1_ + 0.000934*x*_2_ + 0.017923*x*_3_ + 0.006561*x*_4_ + 0.002443*x*_5_ − 0.001710*x*_6_ + 0.003456*x*_7_ − 0.000827*x*_8_ + 0.000460*x*_9_ − 0.000257*x*_10_ + 0.005686*x*_11_ + 0.001614*x*_12_ − 0.001372*x*_13_ + 0.007149*x*_14_ + 0.008494*x*_15_ − 0.001897*x*_16_ + 0.001457*x*_17_ + 0.004286*x*_18_ + 0.002549*x*_19_ + 0.002423*x*_20_ + 0.001829*x*_21_ + 0.002505*x*_22_ − 0.000784*x*_23_ + 0.002143*x*_24_ + 0.005473*x*_25_ + 0.005219*x*_26_ + 0.001997*x*_27_ + 0.006438*x*_28_ + 0.005219*x*_29_ + 0.000965*x*_30_ + 0.002131*x*_31_ + 0.001857*x*_32_ + 0.003832*x*_33_ + 0.005116*x*_34_ + 0.003100*x*_35_ + 0.006076*x*_36_ + 0.003142*x*_37_ + 0.010447*x*_38_ + 0.004462*x*_39_ + 0.007674*x*_40_ + 0.003075*x*_41_.

**Fig. 5:**
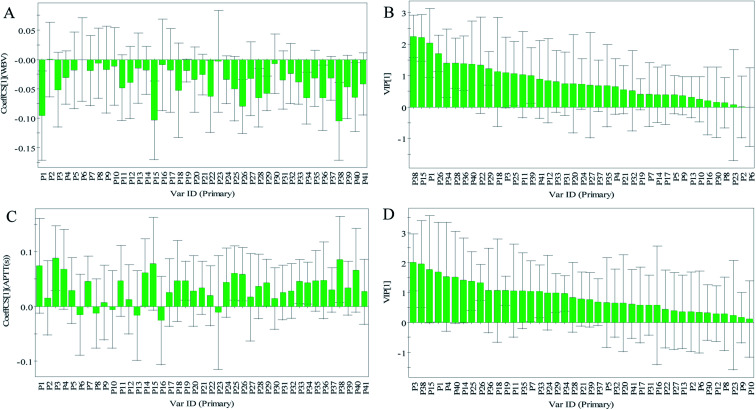
The results of profile–efficacy analysis by partial least squares regression analysis (PLSR). (A) Regression coefficients between 41 common peaks and WBV, (B) regression coefficients between 41 common peaks and APTT, (C) variable importance in projection (VIP) values of 41 common peaks to WBV, and (D) VIP values of 41 common peaks to APTT.

The normalized regression coefficient maps of blood-activation were drawn with the regression equation coefficients (Fig. 5). Except for chromatographic peaks P2 and P6, the other 39 peaks were all negatively related to WBV, indicating that as the levels of compounds represented by these peaks increased, the WBV decreased. Except for peaks P6, P8, P10, P13, P16, and P23, all other peaks were positively related to APTT. In addition, the significance of the *x*-variables for the model could be reflected by the variable importance in projection (VIP) values (VIP > 1.0).^36,37^ Therefore, on one hand, the components corresponding to the peaks P38, P15, P1, P26, P34, P28, P36, P40, P22, P29, P18, P3, P25, P11, and P39 were considered to be closely related to reduced WBV (Fig. 5). On the other hand, the components represented by peaks P3, P38, P15, P1, P4, P40, P14, P25, P26, P36, P18, P19, P11, P35, P7, and P33 were considered to be closely related to prolonged APTT (Fig. 5).

According to the GRA and PLSR results, components represented by peaks P1, P3, P11, P15, P22, P34, P36, P38, and P39 were predicted to be blood-activating. By comparing the chromatograms of the samples with the mixture of reference substances (Fig. 3), peaks P1, P3, P11, P15, P22, P34, P36, P38, and P39 were identified to be salvianic acid A (P1), rutin (P3), ginsenoside Rg_1_ (P11), salvianolic acid B (P15), ginsenoside Rb_1_ (P22), cholic acid (P34), cinobufagin (P36), tanshinone I (P38), and tanshinone IIA (P39), respectively.

### 3.4 Results of verification experiment

**Fig. 6:**
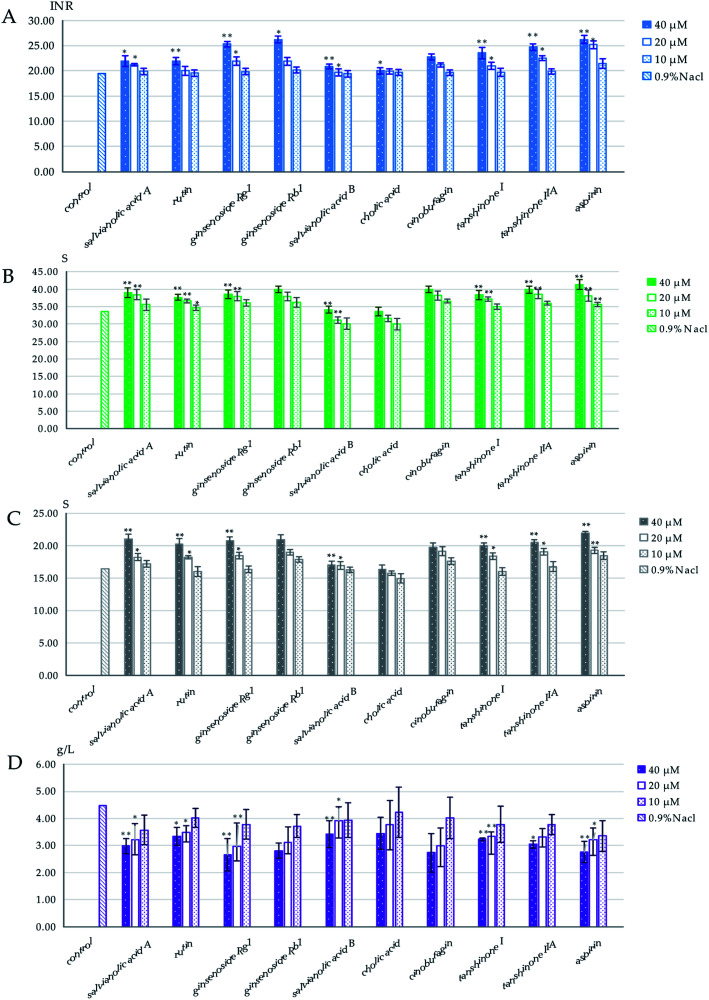
The effects on the coagulation parameters of predicted compounds: (A) PT, (B) TT, (C) APTT, and (D) FIB. Data are expressed as mean ± SD for three independent experiments. Data were analyzed by one-way ANOVA followed by a *post hoc* Dunnett's test. **p < 0.01, *p < 0.05 are significant compared to the control.

The results of the spectrum–effect relationship demonstrated that P1, P3, P11, P15, P22, P34, P36, P38 and P39 might be the blood-activating components in XXT. To further confirm the reliability of the results and determine their dedication to the blood-activating effect of XXT, the anticoagulant effects on APTT, PT, TT, and FIB of normal rat plasma were then tested. As shown in Fig. 6, salvianic acid A (P1), rutin (P3), ginsenoside Rg_1_ (11), ginsenoside Rb_1_ (P22), cinobufagin (P36), tanshinone I (P38), and tanshinone IIA (P39) could increase PT, TT, and APTT and reduce FIB at 40 μM and 20 μM (*p* < 0.05, *p* < 0.01), respectively, but had no obvious effect on these indexes at 10 μM (*p* > 0.05). A certain dose dependence appeared. In addition, the positive control drug, aspirin,^19^ showed a remarkable effect on PT, TT, APTT, and FIB at 40 μM and 20 μM (*p* < 0.05, *p* < 0.01). However, cholic acid (P34) and salvianolic acid B (P15) had no sizeable effect on PT, TT, APTT, or FIB (*p* > 0.05). The results indicated that salvianic acid A, rutin, ginsenoside Rg_1_, ginsenoside Rb_1_, cinobufagin, tanshinone I, and tanshinone IIA showed a significant anticoagulant effect on the rat plasma. As a whole, these seven compounds might play vital roles in the blood-activating effect of XXT.

### 3.5 Network pharmacology

In order to discuss the potential pharmacological mechanisms of XXT, network pharmacology was used based on the seven active components.

#### 3.5.1 Results of construction of networks

##### 3.5.1.1 The compound–target network

**Fig. 7:**
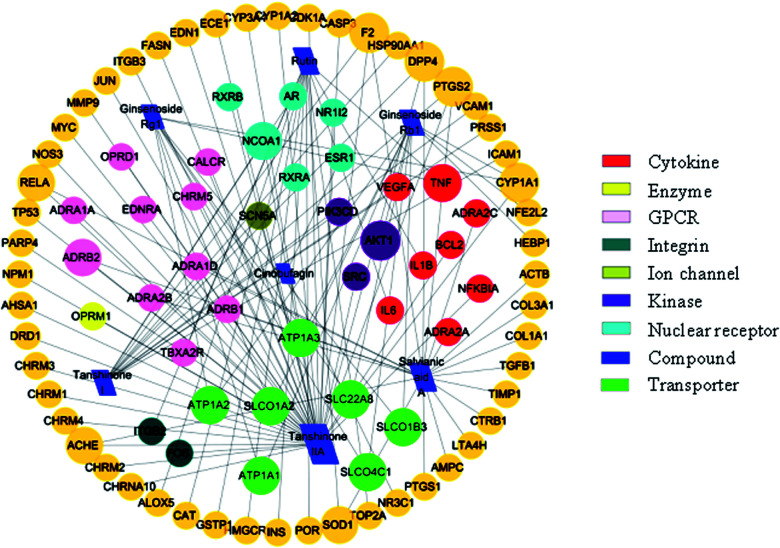
The active compound–target network.

To understand the complex interactions between the seven active compounds and their corresponding targets at a system level, we constructed a network (Fig. 7) based on the active compounds of XXT and their potential targets. This network contained 97 nodes and 111 edges in total. In the network, each node size was proportional to its degree number. The blue parallelograms represent the seven active components, while the circled dots are the targets. Each link represents the interaction between the compound and the target. The targets of tanshinone IIA are greater than 40. The targets of the other four compounds are all greater than 10. The degree values of a node display the number of routes connected to the node. The degree was ranked as tanshinone IIA, salvianic acid A, rutin, tanshinone I, ginsenoside Rb_1_, ginsenoside Rg_1_ and cinobufagin. These targets are composed of eight cytokines, fifty-two enzymes, eleven G-protein-coupled receptors (GPCRs), two integrins, one ion channel, three kinases, six nuclear receptors, and seven transporters. This highlights the critical roles of enzymes, kinase, and transporter.

##### 3.5.1.2 The target–target network

**Fig. 8:**
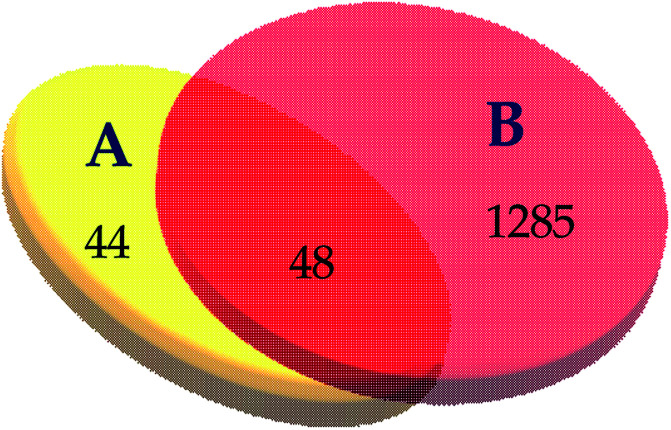
The Venn diagram of active ingredient targets (A) and blood stasis-associated targets (B).

**Fig. 9:**
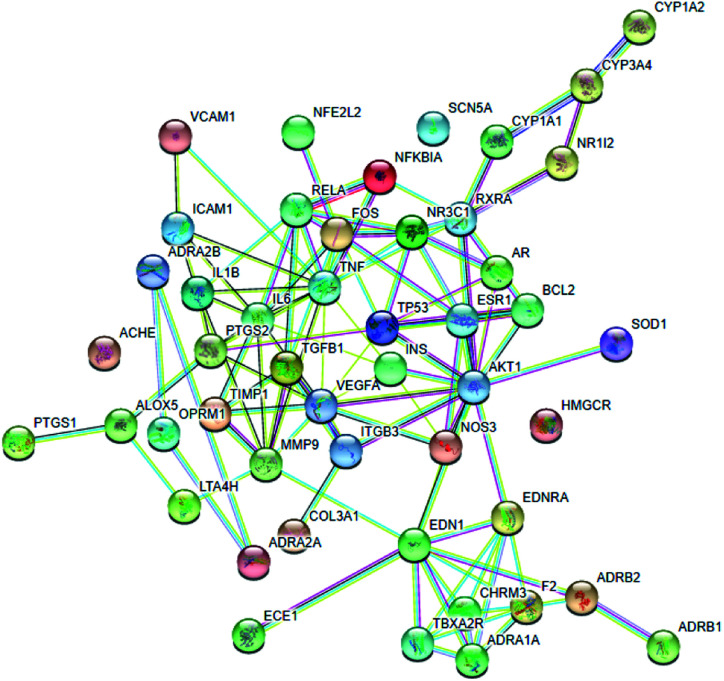
The target–target network from the STRING database.

The blood stasis-associated target proteins were obtained by searching databases. A Venn diagram of 48 core targets was then obtained by blood stasis-associated target intersecting with the above targets of active compounds (Fig. 8). The target–target network was constructed (Fig. 9) from the STRING database by uploading 48 core targets. In the target–target network, each node represents a protein target. Two proteins are linked if they are targeted by the shared component. In the 48 targets, 46 have at least one link to other targets: that is, they share compounds with other targets. A large highly interconnected network, with 46 nodes and 214 edges, was formed by most targets. The top three targets according to degree were TNF, AKT1, and VEGFA. Combined with the betweenness centrality score, AKT1 was selected as a potential key target protein for the treatment of blood stasis.

#### 3.5.2 GO enrichment and KEGG path enrichment analysis

**Fig. 10:**
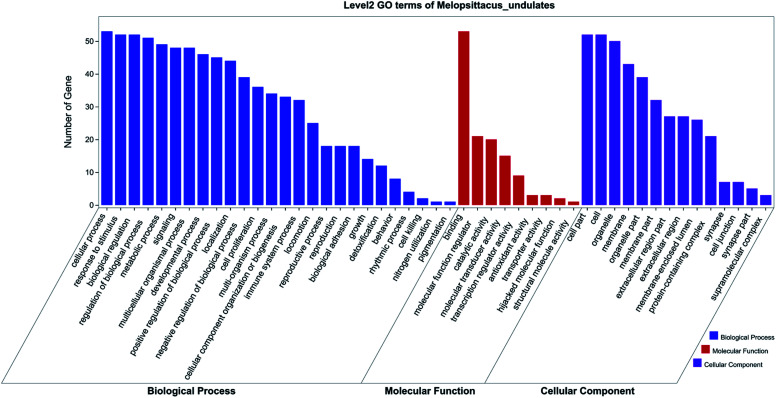
A Gene ontology (GO) enrichment analysis histogram of the active components in XXT.

The gene symbol IDs of the 48 core targets were transformed into 53 gene ensemble IDs with the Ensembl database. The 53 genes were then uploaded to the OmicShare database for Go enrichment analysis. The GO function could be separated into three categories: biological process, molecular function and cellular component. The top three enrichments in the biological process were cellular processes, responses to stimuli, and biological regulation; the top three in the molecular function were binding, molecular function regulation, and catalytic activity; and the top three in the cellular components were cell part, cell, and organelle (Fig. 10).

**Fig. 11:**
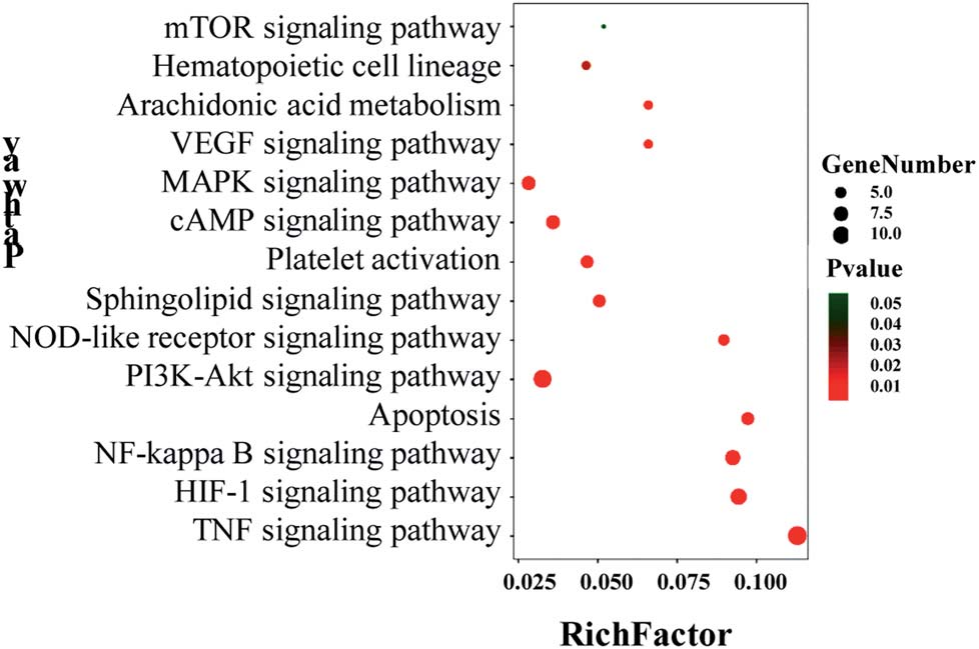
A KEGG enrichment pathway bubble map of the main active components in XXT.

The 48 core targets were uploaded to the DAVID database to obtain 14 pathways related to blood stasis (*p* < 0.05), which were then uploaded to the OmicShare database. A bubble map of the KEGG enrichment pathways of the core targets was obtained (Fig. 11). KEGG analysis demonstrated that multiple pathways were remarkably involved in the mechanism of XXT, including cAMP, TNF, HIF-1, NF-κB, PI3K-Akt, sphingolipid, MAPK, VEGF, platelet activation, apoptosis, arachidonic acid, hematopoietic cell lineage, and NOD-like receptor signaling pathway.

**Fig. 12:**
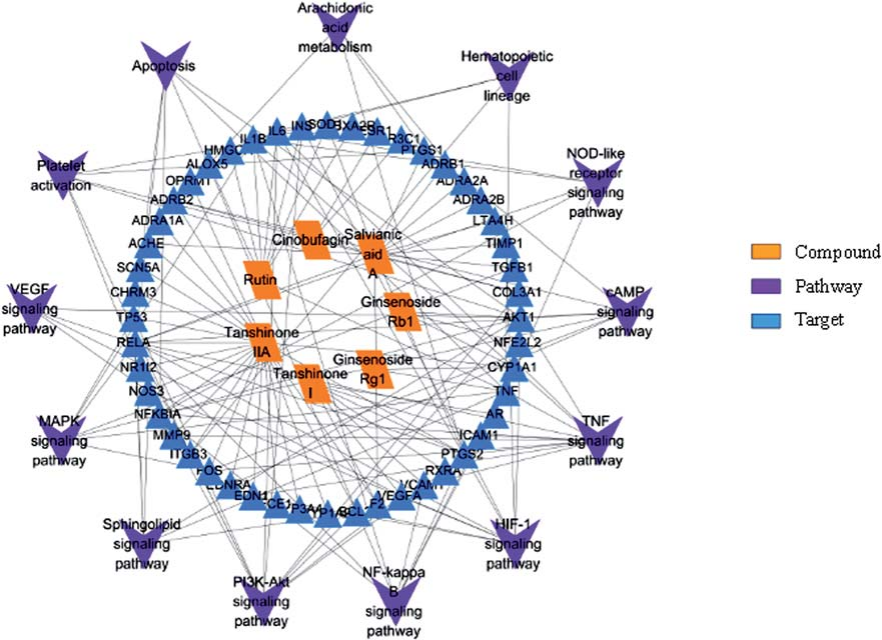
The active compound–target–pathway network map.

Information on pathways and corresponding targets is shown in Table 3. An active compound–target–pathway network map was constructed (Fig. 12).

**Table 3:** The KEGG pathways and corresponding targets in XXT

No.	Pathway	Count	Target name
hsa04024	cAMP signaling pathway	7	EDNRA, AKT1, FOS, ADRB2, ADRB1, RELA, NFKBIA
hsa04668	TNF signaling pathway	12	VCAM1, AKT1, ICAM1, FOS, IL6, TNF, PTGS2, RELA, MMP9, EDN1, NFKBIA, IL1B
hsa04066	HIF-1 signaling pathway	9	AKT1, IL6, INS, RELA, BCL2, EDN1, VEGFA, NOS3, TIMP1
hsa04064	NF-kappa B signaling pathway	8	VCAM1, ICAM1, TNF, PTGS2, RELA, BCL2, NFKBIA, IL1B
hsa04151	PI3K-Akt signaling pathway	11	AKT1, IL6, INS, RELA, RXRA, BCL2, COL3A1, VEGFA, TP53, NOS3, ITGB3
hsa04071	Sphingolipid signaling pathway	6	AKT1, TNF, RELA, BCL2, TP53, NOS3
hsa04010	MAPK signaling pathway	7	AKT1, FOS, TNF, RELA, TP53, IL1B, TGFB1
hsa04370	VEGF signaling pathway	4	AKT1, PTGS2, VEGFA, NOS3
hsa04611	Platelet activation	6	AKT1, COL3A1, PTGS1, TBXA2R, NOS3, ITGB3
hsa04210	Apoptosis	6	AKT1, TNF, RELA, BCL2, TP53, NFKBIA
hsa04150	mTOR signaling pathway	3	AKT1, TNF, INS
hsa00590	Arachidonic acid metabolism	4	PTGS2, PTGS1, LTA4H, ALOX5
hsa04640	Hematopoietic cell lineage	4	IL6, TNF, IL1B, ITGB3
hsa04621	NOD-like receptor signaling pathway	5	IL6, TNF, RELA, NFKBIA, IL1B

## 4 Discussion

The present chemical study has established a valid and satisfactory HPLC method to analyze the fingerprints of 10 kinds of XXT extracts with different polarity solvents. The similarity of the fingerprints (correlation coefficients > 0.815) was acceptable for spectrum–effect relationship analysis, and the coefficients of variance of common peaks (>35%) clearly showed that there was diversity in the contents of the constituents of 10 kinds of XXT extracts. In the pharmacological experiment, hemorheology indexes (such as WBV and PV) and coagulation indicators (PT, TT, APTT, and FIB), the important evaluation indexes in estimating blood stasis,^38,39^ were selected for pharmacodynamic evaluation. Blood stasis plays a vital role in the information process of multiple disorders and diseases.^40,41^ While blood-activation is the essential strategy to ameliorate blood stasis. The results indicated that different batches of XXT exhibited a considerable blood-activating effect on the acute blood stasis model rats. However, significant differences in the levels of WBV (10/s) and APTT between different batches of XXT were found, which might be caused by the different contents of bioactive ingredients in each kind of XXT extract. Therefore, it is necessary to perform a spectrum–effect relationship analysis. Both GRA and PLSR, being widely applied for spectrum–effect relationship analysis of TCM, were used to predict the active components. As a result, nine components with potential strong blood-activating activity were analyzed. In the following pharmacological test, seven compounds, tanshinone I, tanshinone IIA, salvianic acid A, ginsenoside Rg_1_, ginsenoside Rb_1_, rutin, and cinobufagin, were then screened out by an *in vitro* test. Among them, tanshinone I, tanshinone IIA, and salvianic acid A come from *Salvia miltiorrhiza*; ginsenoside Rg_1_, and ginsenoside Rb_1_ come from the total ginsenosides found in ginseng stems and leaves; rutin comes from sophorae flos and cinobufagin comes from bufonis venenum. The pharmacological validation was almost the same as the expected results. In previous reports, the activities of tanshinone I, tanshinone IIA, and salvianic acid A mainly referred to anti-cardiac fibrosis,^42^ anti-tumor,^43,44^ anti-atherosclerosis,^45^ and anti-heart damage effects, and ameliorating endothelial dysfunction,^46^ suppressing excessive oxidation damage and cell apoptosis, anti-blood stasis, and reducing intracellular calcium ion overload,^47^ possessing anticoagulant,^48^ anti-inflammatory,^49^ reducing nerve cell apoptosis and protecting against ischemia/reperfusion injury^50^ and anti-carotid atherosclerosis plaques.^51^ The antiplatelet aggregation activity of ginsenoside Rg_1_ and Rb_1_ has been reported.^52^ In addition, they have activities in treating cardiovascular diseases^53^ and ischemic stroke,^54^ and have anti-inflammatory^55^ and neuroprotective effects against cerebral ischemia.^56^ Rutin had anti-oxidant,^57^ anti-carcinogenic,^58^ liver protection,^59^ anti-inflammatory,^60^ neuroprotective,^61^ and vasoprotective properties.^62^ Cinobufagin inhibited PC3 cell growth^63^ and had antifibrosis properties.^64^ The investigation in the present study showed that “spectrum–effect relationship analysis” is an effective method for looking for the active components in TCM.

To make a further primary prediction of the multiple mechanisms of the effects of XXT, network pharmacology was used to construct an entire drug–target–pathway interaction network in the last part of the present study. The seven screened active components were used as “candidate compounds” for the analysis. The results showed that the main kinds of targets involved in the blood-activating effect were enzymes, kinase, and transporter. The top three targets according to degree, TNF, AKT1, and VEGFA, were considered as potential key target proteins for the treatment of blood stasis in the present study. Moreover, the TNF, NF-κB, and PI3K-Akt signaling pathways were also predicted to be involved in the anti-blood stasis effect of XXT. In previous reports, AKT1, one of three closely related serine/threonine–protein kinases (AKT1, AKT2, and AKT3), is activated reliant on the P13K pathway and is a key node in the signal network, which controls many aspects regarding cell metabolism, proliferation, apoptosis, angiogenesis and anti-hematologic malignancies.^65–67^ TNF (tumor necrosis factor), an inflammatory cytokine (including TNF-α and TNF-β), mainly secreted by macrophages and T lymphocytes during acute inflammation, acts as an inflammatory mediator by preventing endotoxin shock, and has anti-tumor effects and anti-infective effects by inhibiting viral replication and killing virus-infected cells/tumor cells.^68^ VEGFA, a key vascular endothelial growth factor to promote angiogenesis and axonal regeneration,^69^ promotes endothelial cell proliferation, induces cell migration, induces permeabilization of blood vessels, and suppresses apoptosis.^70^ Although further experimental validations of the network pharmacology prediction are required in future studies, utilizing network pharmacology in TCM research is an efficient method to elucidate how multiple components interact with multiple targets, which is consistent with the theory of TCM synergy.

## 5 Conclusions

In the present study, we quickly and accurately screened seven active components from Xueshuan Xinmaining Tablet using a comprehensive evaluation system combining chemical analysis, biological activity evaluation and mechanism study. Firstly, the spectrum–effect relationship was established by linking the peaks in the fingerprints of XXT with the blood-activating bioactivity to screen nine potential active components in XXT. Then, pharmacological tests verified seven of the nine compounds, salvianic acid A (P1), rutin (P3), ginsenoside Rg_1_ (P11), ginsenoside Rb_1_ (P22), cinobufagin (P36), tanshinone I (P38), and tanshinone IIA (P39), and had an anticoagulant effect *in vitro*. Finally, the putative mechanism of these seven components was further discussed using network pharmacology analysis, and three signal pathways (the TNF, NF-κB, and PI3K-Akt signaling pathways) that might be related to anti-blood stasis were found. The study provided a new approach and ideas for the exploration of the active components of TCM.

## Author contributions

Conceptualization: Jing Tan and Jinping Liu; data curation: Hongqiang Lin, Zhongyao Wang, Hanrui Si and Yutong Zhang; formal analysis: Han Wang; funding acquisition: Pingya Li; investigation: Han Wang and Ying Zhang; methodology: Han Wang; project administration: Junli Liu; resources: Ying Zhang; software: Hongqiang Lin; supervision: Junli Liu and Jinping Liu; validation: Kai Sun; visualization: Hanrui Si and Yutong Zhang; writing–original draft: Jing Tan; writing–review & editing: Jinping Liu and Kai Sun.

## Conflicts of interest

The authors declare that there are no conflicts of interest.

## Supplementary Material
